# Glycemic control and body mass index (BMI) as risk factors for erectile dysfunction among Saudi men with diabetes: a systematic review and meta-analysis

**DOI:** 10.1097/MS9.0000000000003917

**Published:** 2025-09-15

**Authors:** Ahmad Salah Alkathiri, Abdullah Khalid Alafif, Osama M. Hayat Nour, Ibraheem Altamimi, Muhammad Anwar Khan, Mohamed Osman Elamin, Rakan Ekram

**Affiliations:** a Department of Health Promotion and Education, Faculty of Public Health and Health Informatics, Umm Al-Qura University, Kingdom of Saudi Arabia; b Family Medicine Academy – Primary Healthcare, Makkah Health Cluster, Makkah, Kingdom of Saudi Arabia; c College of Medicine, King Saud University, Riyadh, Kingdom of Saudi Arabia; d Department of Medical Education, College of Medicine, King Saud bin Abdulaziz University for Health Sciences, Jeddah, Kingdom of Saudi Arabia; e King Abdullah International Medical Research Center, Jeddah, Kingdom of Saudi Arabia; f Department of Environmental Health, Faculty of Public Health and Health Informatics, Umm Al-Qura University, Makkah, Kingdom of Saudi Arabia; g Health Administration and Hospitals Department, Faculty of Public Health and Health Informatics, Umm Al-Qura University, Makkah, Kingdom of Saudi Arabia

**Keywords:** body mass index, diabetes mellitus, erectile dysfunction, glycemic control, meta-analysis, Saudi Arabia

## Abstract

**Background:**

Erectile dysfunction (ED) is a prevalent complication among diabetic men, impacting their quality of life and overall health outcomes. Both glycemic control and body mass index (BMI) have been implicated as potential risk factors for ED in this population, but the evidence remains inconclusive, particularly in the context of Saudi Arabia.

**Study Aim:**

This meta-analysis aimed to systematically evaluate the association between glycemic control, BMI, and the prevalence of ED among diabetic Saudi men.

**Methods:**

A comprehensive literature search was conducted in major electronic databases to identify relevant studies published up to 31 December 2023. Eligible studies were cross-sectional or prospective in design, included male participants diagnosed with diabetes mellitus, and reported data on glycemic control, BMI, and ED prevalence. Data were extracted, and a random-effects model was used to calculate pooled odds ratios (ORs) with 95% confidence intervals (CIs). Heterogeneity was assessed using the *I*^2^ statistic, and publication bias was evaluated using funnel plots.

**Results:**

Five studies met the inclusion criteria, comprising a total sample of 1710 diabetic Saudi men. The pooled analysis revealed a significant association between uncontrolled diabetes mellitus and increased risk of ED (OR = 3.01, 95% CI: 0.98–9.26), with substantial heterogeneity (*I*^2^ = 92%). However, no significant association was found between high BMI (overweight or obese) and ED prevalence (OR = 0.73, 95% CI: 0.34–1.56), with high heterogeneity (*I*^2^ = 85%). Funnel plots suggested minimal publication bias.

**Conclusion:**

Our findings highlight the critical role of glycemic control in determining ED risk among diabetic Saudi men. While uncontrolled diabetes appears to significantly increase the likelihood of ED, BMI alone may not be a strong predictor in this population. Clinicians should prioritize aggressive management of diabetes to mitigate ED risk, while individualized approaches to weight management may be warranted for overall health optimization.

## Introduction

Erectile dysfunction (ED) is a common and distressing condition characterized by the inability to achieve or maintain an erection sufficient for satisfactory sexual performance^[1]^. While ED can occur in men of all ages, it becomes more prevalent with advancing age and is often associated with various comorbidities, including diabetes mellitus (DM)^[2]^. The interplay between diabetes and ED is well established, with diabetes being recognized as one of the leading risk factors for ED worldwide^[1,3]^. The burden of diabetes-related ED is particularly pronounced in Saudi Arabia, where diabetes prevalence rates have reached epidemic proportions, with an estimated prevalence of 32.4% among adults aged 55 years or more^[4]^.

The pathophysiological mechanisms underlying diabetes-related ED are multifactorial and complex. Chronic hyperglycemia, a hallmark feature of diabetes, leads to microvascular damage and endothelial dysfunction, compromising penile blood flow and impairing the erectile response^[5,6]^. Additionally, diabetes-associated neuropathy and autonomic dysfunction contribute to the development of ED by disrupting the intricate neural pathways involved in erectile physiology^[6]^. Psychological factors such as depression, anxiety, and diabetes-related distress further exacerbate the burden of ED in diabetic men, contributing to a vicious cycle of sexual dysfunction and impaired quality of life^[7]^.

In addition to glycemic control, obesity has emerged as a significant modifiable risk factor for ED in the general population^[8,9]^. The adverse effects of obesity on erectile function are thought to result from a complex interplay of metabolic, hormonal, and vascular factors^[10]^. Excess adiposity is associated with systemic inflammation, insulin resistance, and dyslipidemia, all of which contribute to endothelial dysfunction and impaired penile vascular perfusion^[11,12]^. Moreover, obesity-related comorbidities such as hypertension, cardiovascular disease, and metabolic syndrome further compound the risk of ED, highlighting the intricate relationship between obesity and erectile health^[13]^.

Despite the well-established individual associations between diabetes, obesity, and ED, the precise interplay between glycemic control, body mass index (BMI), and erectile function in diabetic populations remains incompletely understood, particularly among Saudi men. While some studies have suggested that poor glycemic control and obesity independently increase the risk of ED among diabetic individuals^[14–16]^, others have reported conflicting findings or observed no significant association^[14]^. This discrepancy underscores the need for a comprehensive synthesis of the existing literature to elucidate the collective impact of glycemic control and BMI on ED prevalence among diabetic Saudi men.

Moreover, the cultural, social, and healthcare context of Saudi Arabia may further influence the relationship between diabetes, obesity, and ED. Cultural norms surrounding sexuality and barriers to seeking healthcare may impact the reporting and management of ED in this population^[17]^. Additionally, disparities in healthcare access and utilization, as well as variations in diabetes management practices, may contribute to differences in ED prevalence and outcomes among diabetic Saudi men^[17]^.

The primary aim of this meta-analysis is to systematically review and quantitatively synthesize the existing evidence on the relationship between glycemic control and BMI as risk factors for ED among diabetic men in Saudi Arabia. This study seeks to identify the prevalence of ED among diabetic men with different BMI categories (normal weight, overweight, and obese) and compare the prevalence of ED in men with controlled versus uncontrolled DM.

## Methodology

### Study design

The reporting of this meta-analysis follows the guidelines set forth by the Preferred Reporting Items for Systematic Reviews and Meta-Analyses (PRISMA) to enhance transparency and reproducibility. The PRISMA checklist was used to ensure the completeness of reporting throughout the study^[18]^.

### Search strategy

The literature search was designed to identify all relevant studies that investigated the association between glycemic control, BMI, and ED in diabetic Saudi men. We conducted a thorough search of electronic databases including PubMed, Scopus, Web of Science, and Embase. The search strategy combined terms related to “erectile dysfunction,” “diabetes mellitus,” “glycaemic control,” “Body Mass Index,” and “Saudi Arabia” without language or publication date restrictions. The search strings were adapted to each database’s syntax and filters. Additionally, the reference lists of included studies and relevant reviews were manually searched to identify further studies not captured by the database searches.

**HIGHLIGHTS**

- The meta-analysis includes five studies comprising a total of 1710 diabetic men from Saudi Arabia.
- Uncontrolled diabetes significantly elevates the risk of erectile dysfunction (ED) (odds ratio = 3.01).
- There is no prominent correlation observed between high BMI and the prevalence of ED.
- The study highlights the significance of pre-emptive management of diabetes to mitigate the ED risk.
- Results suggest that body mass index alone might not be a strong predictor of ED in the Saudi population.
- The current findings advocate for a subtler strategy to address ED among male diabetic patients.

### Inclusion and exclusion criteria

Studies were included if they met the following criteria: (1) cross-sectional or prospective design, (2) included men diagnosed with DM, (3) reported on the prevalence of ED, (4) assessed the relationship between ED and either glycemic control or BMI, and (5) conducted in Saudi Arabia. We excluded studies that (1) did not report specific data for the Saudi population, (2) were case reports, letters, comments, or reviews, and (3) lacked clear definitions or assessments of ED, glycemic control, or BMI.

### Data extraction

Two independent reviewers extracted data from the included studies using a standardized data extraction form. Extracted information included study characteristics (author, year of publication, study design, and setting), population characteristics (sample size, age, and diabetes type), and key findings related to the prevalence of ED among different BMI categories and glycemic control statuses. Any discrepancies between reviewers were resolved through discussion or consultation with a third reviewer.

### Statistical analysis

The association between glycemic control (uncontrolled vs. controlled DM) and ED, and the association between BMI (overweight/obese vs. normal weight) and ED were quantified using odds ratios (ORs) with 95% confidence intervals (CIs). A random-effects model was utilized to account for potential heterogeneity across studies. Heterogeneity among the studies was assessed using the *I*^2^ statistic, with values of 25%, 50%, and 75% indicating low, moderate, and high heterogeneity, respectively. Publication bias was evaluated through visual inspection of funnel plots. Sensitivity analyses were conducted to explore the impact of individual studies on the overall results. All statistical analyses were performed using Review Manager (RevMan), version 5.4, The Cochrane Collaboration, 2020.

## Results

### Search results

**Figure 1.**
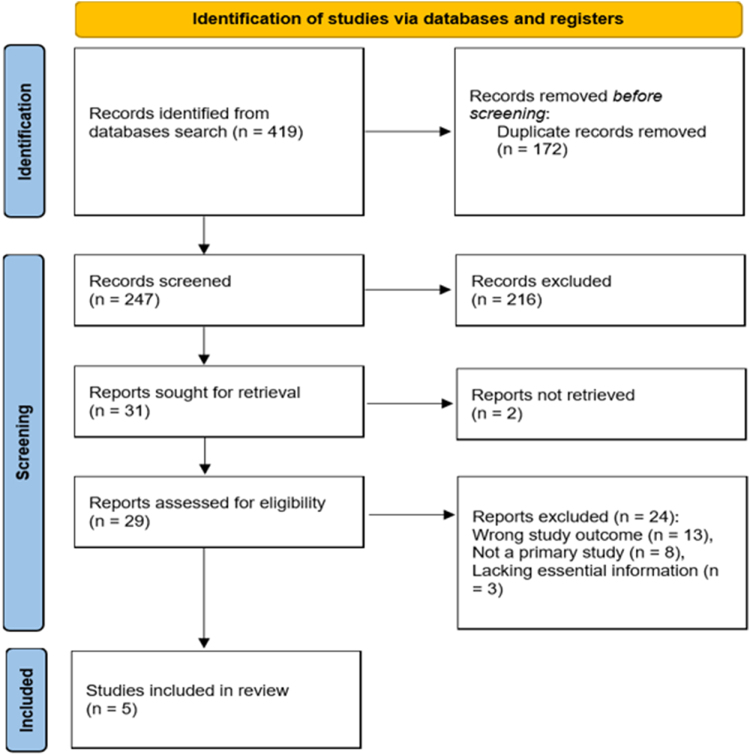
PRISMA flow diagram for the study search and selection processes.

The initial database search yielded 419 potentially relevant studies. Following the removal of duplicates, title and abstract screening, and full-text assessment, a total of 5 studies were included in the quantitative synthesis^[19–23]^. The PRISMA flow diagram illustrates the study selection process (Fig. 1).

### Characteristics of included studies

In the review, five studies were included for quantitative data synthesis. These studies, conducted between 2003 and 2020, offer a comprehensive overview of the prevalence of ED among diabetic men in Saudi Arabia, emphasizing the potential impact of BMI and glycemic control (Table 1).

**Table 1:** Characteristics and findings of the included studies (*n* = 5)

Study	City	Study design	Population type	Study year	Diagnostic tool	Number of participants	Prevalence of ED among overweight men	Prevalence of ED among obese men	Prevalence of ED among men with normal BMI	Prevalence of ED among men with uncontrolled DM	Prevalence of ED among men with controlled DM
Almigbal, 2019^[19]^	Riyadh	Cross-sectional	Men with T2DM	2017	IIEF	293	79.8%	78.5%	88.2%	87.7%	71.8%
Almogbel, 2014^[20]^	Riyadh	Cross-sectional	Men with diabetes mellitus registered in primary care clinics	2012–2013	IIEF	376	77.9%	81.1%	97.0%	84.7%	78.7%
Al-Turki, 2007^[21]^	Riyadh	Cross-sectional	Men with diabetes mellitus attending a primary care clinic	2005–2006	IIEF	186	76.9%	78.4%	71.4%	80.0%	75.5%
Batais *et al*, 2020^[22]^	Riyadh	Cross-sectional	Men with T2DM	2017	IIEF	293	79.8%	78.5%	88.2%	NR	NR
El-Sakka and Tayeb, 2003^[23]^	Makkah	Prospective study	Men with noninsulin-dependent diabetes	2001–2002	IIEF	562	81.9%	91.9%	70.0%	95.3%	58.6%

The cities of Riyadh and Makkah served as the geographical focal points for this investigation. Notably, all but one study was carried out in Riyadh, highlighting a concentration of research interest in the capital region^[19–22]^. In contrast, El-Sakka and Tayeb^[23]^ conducted their prospective study in Makkah, offering a broader geographical perspective within the kingdom.

Regarding study design, the majority of the research adopted a cross-sectional approach^[19–22]^, which is instrumental in understanding the prevalence and distribution of ED in specific populations at a given time. The exception was the study by El-Sakka and Tayeb^[23]^, which employed a prospective design, providing insights into the progression and potentially causal relationships over time.

The population type across these studies was consistently men with DM, with a specific focus on those with type 2 DM (T2DM) in three studies^[19,22,23]^, reflecting the global burden of this disease and its implications on sexual health. The participant numbers varied, ranging from 186 in the study by Al-Turki^[21]^ to 562 in the work of El-Sakka and Tayeb^[23]^, indicating a wide range in the scale of research efforts.

The International Index of Erectile Function (IIEF) was universally applied as the diagnostic tool across all studies, ensuring a consistent and reliable measure of ED among participants. This uniformity in assessment methodology enhances the comparability of results across the studies included in this meta-analysis.

The findings revealed a nuanced picture of ED prevalence in relation to BMI and glycemic control. For overweight men, the prevalence of ED ranged from 76.9% to 81.9%, while for obese men, it was slightly higher, with figures between 78.4% and 91.9%. Interestingly, the prevalence of ED among men with a normal BMI was reported in a narrower range, from 70.0% to 97.0%, suggesting that while BMI is a significant factor, other variables also play crucial roles in the development of ED among diabetic men.

Regarding glycemic control, the prevalence of ED among men with uncontrolled DM was notably high, peaking at 95.3% in the study conducted by El-Sakka and Tayeb^[23]^. Conversely, men with controlled DM exhibited a lower prevalence, with figures down to 58.6%, underscoring the critical impact of glycemic management on sexual health.

### Glycemic control and ED

**Figure 2.**
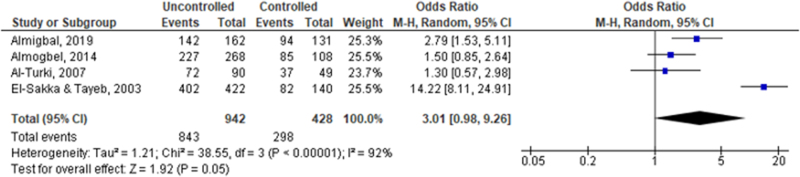
Forest plot of glycemic control as a risk factor for erectile dysfunction among diabetic Saudi men.

The forest plot (Fig. 2) provided a visual and statistical synthesis of the relationship between glycemic control and ED among the studied population. The pooled OR for the prevalence of ED among men with uncontrolled diabetes compared to those with controlled diabetes was 3.01 (0.98, 9.26), indicating a trend toward a higher risk of ED among men with poor glycemic control, although the wide CI reflects substantial heterogeneity (*I*^2^ = 92%) and suggests caution in interpretation.

Notably, the study by El-Sakka and Tayeb^[23]^ reported the most pronounced effect [OR = 14.22 (8.11, 24.91)], significantly influencing the overall estimate. The high degree of heterogeneity and the borderline significance of the overall effect test (*P* = 0.05) underscore the complex interplay between diabetes management and erectile function, as well as the variability across different populations and study methodologies. Sensitivity analysis was performed to explore potential sources of heterogeneity and by excluding one study at a time, we found that by excluding the study of El-Sakka and Tayeb^[23]^, the pooled estimate was OR = 1.82 (1.15, 2.90), and there was no longer significant heterogeneity (*I*^2^ = 34%, *P* = 0.22).

**Figure 3.**
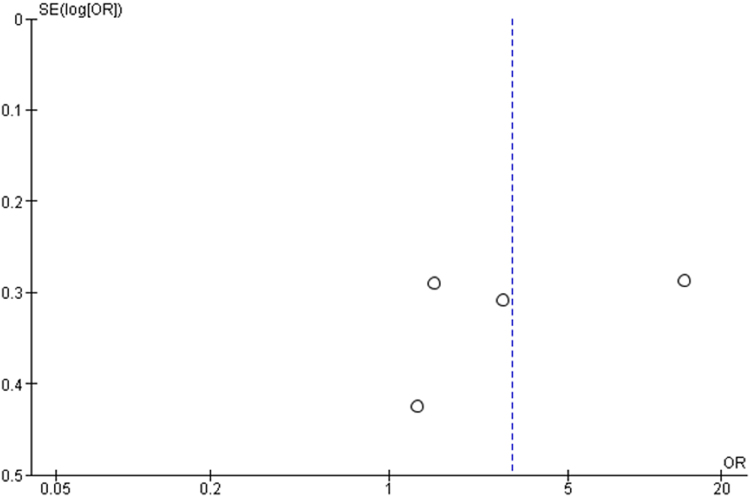
Funnel plot of assessment of publication bias for BMI data.

The funnel plot (Fig. 3) assessing publication bias for glycemic control data revealed a symmetrical distribution, suggesting a low risk of publication bias and increasing confidence in the meta-analysis findings.

### BMI and ED

**Figure 4.**
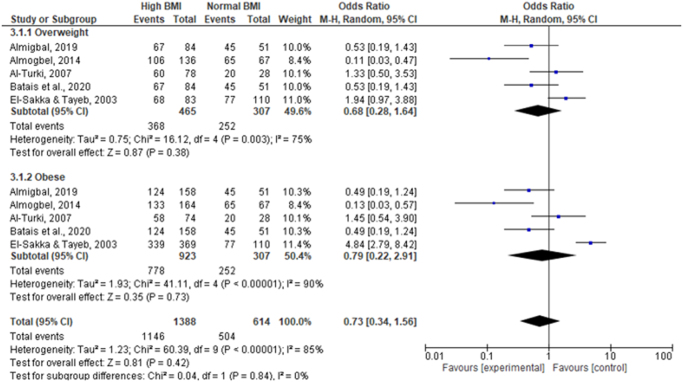
Forest plot of obesity and overweight as risk factors for erectile dysfunction among diabetic Saudi men.

The analysis of the impact of BMI on ED, as visualized in the forest plot (Fig. 4), was segmented into overweight and obese subgroups. Neither subgroup showed a statistically significant association with ED when compared to men with a normal BMI, with pooled ORs of 0.68 (0.28, 1.64) for overweight and 0.79 (0.22, 2.91) for obese men. These findings indicate no clear evidence of an increased risk of ED associated with higher BMI categories in this population. However, the considerable heterogeneity (*I*^2^ = 75% for overweight and *I*^2^ = 90% for obese subgroups) suggests that factors beyond BMI may influence ED prevalence among diabetic men.

The overall pooled analysis of high BMI (combining overweight and obese subgroups) compared to normal BMI yielded an OR of 0.73 (0.34, 1.56), with an *I*^2^ of 85%, further confirming the lack of significant association between BMI and ED in the context of diabetes. The test for subgroup differences did not reveal significant variation between overweight and obese groups (*P* = 0.84), reinforcing the consistent lack of association across different levels of BMI.

**Figure 5.**
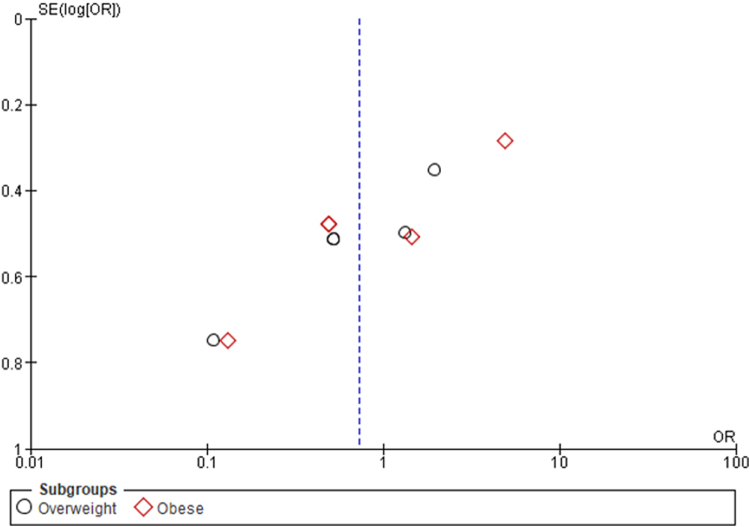
Funnel plot of assessment of publication bias for BMI data.

The funnel plot (Fig. 5) for BMI data demonstrated a symmetrical distribution, indicative of minimal publication bias in this subset of the meta-analysis. This symmetry supports the reliability of the analysis, suggesting that the findings are reflective of the available evidence without significant skewing by unpublished data or selective reporting.

## Discussion

Our meta-analysis critically evaluated the association between glycemic control, BMI, and the prevalence of ED among diabetic men in Saudi Arabia. Through a detailed examination of five studies, we found a pronounced trend suggesting that uncontrolled DM significantly correlates with an increased risk of ED, while the influence of BMI on ED prevalence among diabetic men appeared to be less definitive.

The pooled analysis indicated a threefold increase in the risk of ED among men with uncontrolled diabetes compared to those with controlled diabetes, with a pooled OR of 3.01 (0.98, 9.26). This finding, though marginally crossing the threshold of statistical significance, underscores the critical role of glycemic control in the sexual health of diabetic men. It aligns with the study by El-Sakka and Tayeb^[23]^, which reported a markedly high OR [OR = 14.22 (8.11, 24.91)], emphasizing the severe impact uncontrolled diabetes can have on erectile function.

These results resonate with existing literature that consistently links poor glycemic control with microvascular complications, which are thought to underpin the pathogenesis of ED in diabetic populations^[3,24–26]^. Hyperglycemia-induced oxidative stress, endothelial dysfunction, and autonomic neuropathy are pivotal mechanisms that impair nitric oxide synthesis and action, crucial for erectile physiology^[27]^. Moreover, the psychological burden of diabetes management may also contribute to ED, suggesting a multifactorial etiology encompassing both organic and psychogenic elements^[28]^.

The significant heterogeneity observed in our analysis (*I*^2^ = 92%) could be attributed to variations in study populations, diabetes duration, and differences in glycemic control assessment methods. This variability highlights the complexity of diabetes management and its impact on sexual health, suggesting that individualized approaches to glycemic control may be necessary to mitigate ED risk effectively.

Contrary to expectations, our analysis did not find a significant association between BMI and ED among diabetic men. The pooled ORs for overweight and obese men compared to those with normal BMI were 0.68 (0.28, 1.64) and 0.79 (0.22, 2.91), respectively, indicating no clear evidence of increased ED risk in these groups. This finding diverges from the general population, where obesity is a well-established risk factor for ED, likely due to its association with cardiovascular disease, hypertension, and metabolic syndrome, all of which are pathogenic mechanisms shared with ED^[9,16,29]^.

Several factors may explain this discrepancy in diabetic men. First, the pathophysiological impact of diabetes on erectile function might overshadow the influence of BMI. Diabetes-induced endothelial dysfunction and autonomic neuropathy could be so profound that the additional burden of obesity does not significantly alter the risk profile for ED. Second, the adipose tissue in obese individuals secretes a variety of bioactive substances, including adipokines, which may have both detrimental and protective effects on vascular health^[30,31]^. The net impact of these factors on erectile function in the context of diabetes remains to be fully elucidated.

Furthermore, the high heterogeneity observed in the analysis of BMI and ED (*I*^2^ = 85%) suggests that other variables, such as physical activity, diet, and genetic predispositions, may interact with BMI to influence ED risk. These factors were not uniformly reported or controlled for in the included studies, potentially contributing to the observed variability.

### Clinical implications and future directions

Our findings have significant clinical implications for the management of diabetic men with or at risk for ED. They highlight the paramount importance of stringent glycemic control as a primary intervention to prevent or mitigate ED in this population. Clinicians should prioritize comprehensive diabetes management strategies that encompass lifestyle modifications, pharmacotherapy, and regular monitoring to optimize glycemic control^[32]^.

Given the less clear role of BMI in influencing ED risk in diabetic men, our results suggest that weight management interventions should be recommended primarily for their well-established benefits in improving overall diabetes control and reducing cardiovascular risk, rather than specifically for ED prevention. However, given the complexity of the relationship between obesity, diabetes, and ED, individualized assessment and counseling regarding weight management may still be beneficial for some patients^[33]^.

Future research should aim to unravel the intricate interplay between diabetes, BMI, and ED by incorporating longitudinal designs, standardized measures of glycemic control and ED assessment, and comprehensive evaluation of potential confounders and mediators, including lifestyle factors, psychosocial variables, and molecular biomarkers. Such studies could provide deeper insights into the pathophysiological mechanisms linking diabetes and obesity with ED and inform more targeted interventions.

Moreover, exploring the psychological dimensions of ED in diabetic men, including the impact of diabetes distress and depression, could yield important insights into holistic management approaches that address both the physical and emotional aspects of ED.

## Conclusion

In conclusion, our meta-analysis underscores the critical role of glycemic control in the sexual health of diabetic men and suggests that the impact of BMI on ED prevalence in this population may be less significant than previously thought. These findings call for a nuanced approach to managing ED in diabetic men, emphasizing the importance of comprehensive diabetes care and highlighting the need for further research to fully understand the multifactorial nature of ED in this population.

## Data Availability

All data relevant to the study are included in the article.

## Research registration unique identifying number (UIN)

Approved by PROSPERO (Registration Number: CRD42024548540).
